# A national HIV clinical mentorship program: Enabling Zambia to accelerate control of the HIV epidemic

**DOI:** 10.1371/journal.pgph.0000074

**Published:** 2022-02-23

**Authors:** Mary Adetinuke Boyd, Sombo Fwoloshi, Peter A. Minchella, James Simpungwe, Terence Siansalama, Danielle T. Barradas, Minesh Shah, Lloyd Mulenga, Simon Agolory

**Affiliations:** 1 U.S. Centers for Disease Control and Prevention, Atlanta, Georgia, United States of America; 2 Ministry of Health, Lusaka, Government of the Republic of Zambia; UP Manila: University of the Philippines Manila, PHILIPPINES

## Abstract

Although Zambia has increased the proportion of people living with HIV (PLHIV) who are on antiretroviral therapy (ART) in recent years, progress toward HIV epidemic control remains inconsistent. Some districts are still failing to meet the UNAIDS 90/90/90 targets where 90% of PLHIV should know their status, 90% of those diagnosed should be on ART, and 90% of those on ART should achieve viral load suppression (VLS) by 2020. Providing consistently excellent HIV services at all ART health facilities is critical for achieving the UNAIDS 90/90/90 targets and controlling the HIV epidemic in Zambia. Zambia Ministry of Health, in collaboration with the U.S. Centers for Disease Control and Prevention (CDC), aimed to achieve these targets through establishing a national HIV clinical mentorship program in which government-employed mentors were assigned to specific facilities with a mandate to identify and ameliorate programmatic challenges. Mentors were hired, trained and deployed to individual facilities in four provinces to mentor staff on quality HIV clinical and program management. The pre-mentorship period was July 2018–September 2018 and the post-mentorship period was July 2019–September 2019. Review of key programmatic indicators from the pre and post-deployment periods revealed the proportion of people who had a positive HIV test result out of those tested increased from 4.2% to 6.8% (P <0.001) as fewer HIV tests were needed despite the number of PLHIV being identified and placed on ART increasing from 492,613 to 521,775, and VLS increased from 84.8% to 90.1% (p <0.001). Key considerations in the establishment of an HIV clinical mentorship program include having a government-led process of regular site level data review and continuous clinical mentorship underpinned by quality improvement methodology.

## Introduction

With an estimated 1.2 million people living with HIV (PLHIV) and 12% prevalence, Zambia is severely affected by the HIV epidemic [1]. By the end of September 2018, 892,362 (72%) people living with HIV (PLHIV) in Zambia were on life saving antiretroviral therapy (ART) [2]. Despite improvements in ART coverage, overall progress toward UNAIDS 90-90-90 targets for HIV epidemic control (90% of PLHIV should know their status, 90% of those diagnosed should be on ART, and 90% of those on ART should achieve viral load suppression (VLS) by 2020) is slow and inconsistent. While the factors limiting progress are diverse, an effort to evaluate the provision of services within Zambia’s HIV program revealed that a lack of continuous capacity building and mentorship for healthcare workers from experienced HIV clinicians was a key gap [3]. Due to a strained health workforce, clinical services in Zambia are generally provided by mid-level practitioners such as clinical officers and nurses who often have limited access to in-service training, ongoing continuous medical education or clinical mentorship in the delivery of quality client services [3]. This is compounded by the reality that specialist care is more often concentrated in urban areas, far out of the reach of much of Zambia’s population. In the United States, Canada and Uganda, HIV clinical mentorship has been shown to improve clinical practices and outcomes related to linking and retaining patients in HIV care and across varying disciplines [4–7]. Furthermore, in resource limited settings where distance is a barrier to providing ongoing clinical training and mentorship, Project ECHO (Extension for Community Healthcare Outcomes), which uses video-conferencing technology to bridge knowledge gaps between experts and providers, has demonstrated an impact on patient outcomes [8–10]. In an evaluation of Project ECHO in Namibia, nurses who provide majority of the HIV service delivery reported increased HIV clinical knowledge and self-efficacy by adopting this method of telementoring [10]. Thus, the Zambian Ministry of Health, with support from U.S. Centers for Disease Control and Prevention (CDC), established a national HIV clinical mentorship program with the aim of improving performance across key HIV program indicators. Improved case finding, increasing the number of PLHIV on treatment and viral load suppression were particular areas of focus.

## Materials and methods

### Study design and setting

**Fig 1:**
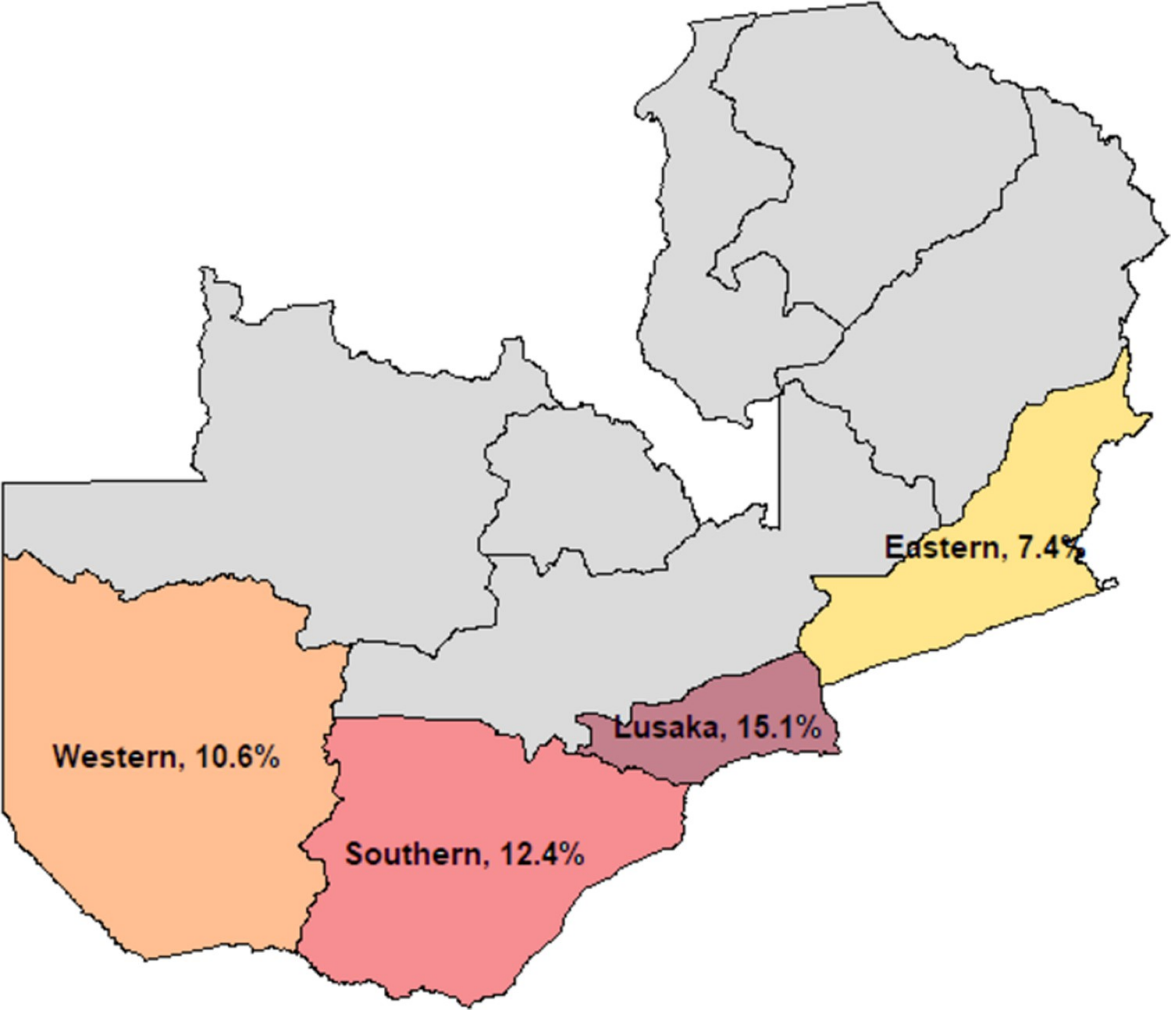
Map of Zambia with HIV prevalence among adults aged 15–49 by province. Labels indicate the provinces in which the HIV clinical mentorship program was implemented [11].

We conducted a retrospective pre-post analysis of routine program data collected during two periods: July–September 2018 and July–September 2019. The HIV clinical mentorship program was implemented across four of Zambia’s ten provinces, Eastern, Western, Southern, and Lusaka, which enabled the program to reach a wide array of clinical care settings (e.g., urban, rural, semi-urban) and geographies with varying HIV prevalence (Fig 1).

### Program

Starting in October 2018, 27 mentors were hired, trained, and deployed to 45 districts with high HIV burdens across the four selected provinces. Mentors had a variety of clinical backgrounds (nurses, clinical officers, and medical officers), were hired through a competitive process, and participated in a two-week training focused on the fundamentals of HIV clinical and program management. Following training, each mentor was assigned 2–10 health facilities, each of which employed more than 15 health workers and managed patient volumes ranging from 500 to 12,000 clients on ART. In general, each mentor was assigned to one district, though there were exceptions in which mentors were tasked with covering multiple districts or multiple mentors were assigned to facilities within the same district. Deployment of mentors and assignment of health facilities was done strategically in order to leverage interconnectedness. Specifically, more senior mentors were assigned to cover all districts that did not have a dedicated mentor and to perform visits supplemented with telephone calls with the same frequency as districts with a dedicated mentor. Mentor deployment was also done in collaboration with district health offices to ensure local support for the program. Overall, there were 28 mentor-supported districts that accounted for approximately 83% of the patients on ART within the four selected provinces at the time the program was initiated.

Following their training and subsequent deployment, mentors continued to interact with experienced public health professionals and HIV clinical care providers from CDC, local and international implementing partners and academic institutions, and the Zambia Ministry of Health chief mentor via weekly video conferences, on-site mentoring, and Tele ECHO™ sessions [12]. Weekly Tele ECHO sessions aimed to both build knowledge through presentation and discussion of common or complex HIV cases, and to facilitate interaction among healthcare workers from across different health facilities to help create a community of practice with substantial cross-learning across the network. Additionally, weekly check-ins and on-site mentoring focused on data review using provided reporting templates and implementation of continuous quality improvement methodology. Overall, ongoing interactions with mentors aimed to improve clinical practice by addressing key programmatic barriers to epidemic control and by sharing best practices. Specific areas of focus included mentored use of HIV risk screening tools in outpatient departments and safe and ethical index client elicitation to improve HIV case finding. Mentors implemented the national risk screening tool at facility level and provided facility-based group training and 1:1 mentoring to HIV testers and other facility staff on the proper use of risk screening tool, routinely observing and providing on the spot feedback. To address retention in care, mentors implemented scheduled facility appointments in ART clinics, monitored missed appointments and provided training in patient satisfaction and customer care. Mentors also guided facility staff in the implementation of routine HIV viral load testing and established high viral load clinic days for the management of patients with high HIV viral load. Notably, mentors institutionalized facility mid-week continuous quality improvement meetings where the facility nurse-in-charge leads facility staff in the review and accompanying process improvements of key HIV indicators.

### Outcomes

The outcomes of interest in our analysis were related to HIV testing services (HTS) and HIV viral load (VL) monitoring. Those related to HTS were: 1. the proportion of people who had a positive HIV test result out of those tested per national guidelines; and 2. the proportion of people who had a positive HIV test result out of the total number of index contacts who received HTS and received their test results. Index testing contacts were defined as sexual contacts or biological children of PLHIV who were elicited and offered HTS [13]. Outcomes for HIV viral load testing were: 1. viral load coverage, defined as the number of PLHIV with documented HIV viral load test results during the last 12 months divided by the number of PLHIV eligible for viral load testing, and 2. HIV viral load suppression, defined as the number of PLHIV with viral load test results <1,000 cp/ml during the previous 12 months divided by the number with a HIV viral load test result. For all outcomes we used definitions and guidance from the U.S President’s Emergency Plan For AIDS Relief (PEPFAR) Monitoring, Evaluation and Reporting (MER) Indicator Reference Sheet version 2.3 [13].

### Data source and analysis

De-identified aggregate program data were abstracted from PEPFAR’s Data for Accountability Transparency and Impact (DATIM) system. The pre-mentorship period was July 2018–September 2018 and the post-mentorship period was July 2019–September 2019. Proportions were calculated for outcomes of interest and analyzed using descriptive statistics. Chi-square tests were used to compare proportion of people who had a positive HIV test result out of those tested, viral load coverage, and viral load suppression. All analyses were performed using R (version 4.0.2).

### Ethics approval and consent to participate

Ethical approval for this retrospective analysis of routine de-identified program data was approved by the ERES Converge Zambian Institutional Review Board (IRB) and U.S. Centers for Disease Control and Prevention.

## Results

Given the high proportion of PLHIV represented by mentor-supported districts and the intended impacts of mentors beyond their districts, results from all districts in the four selected provinces were included in the analysis. Between the pre- and post-mentorship periods the proportion of people who had a positive HIV test result out of those tested increased from 4.2% to 6.8% (p<0.001) and this resulted in total of 38,555 PLHIV diagnosed in the post mentorship period compared to 37,206 in the pre-mentorship period. The number of cases identified in the post-mentorship period included 9,091 cases identified through index case testing, an increase from 4,115 and proportion of people who had a positive HIV test result out of the total number of index contacts who received HTS increased from 11.6% to 16.9% (p<0.001) as well as increased contribution of index-case testing to overall case finding from 12% (4,006/33,574) to 26% (9,039/34,944) in one year. Number of PLHIV on ART increased from 492,613 to 521,775 and documented viral load coverage increased from 40.8% to 74.1% (p<0.001) with an increasing rate of viral suppression from 84.8% to 90.1% (p<0.001). Also, number of HIV tests performed decreased from 878,948 to 565,226. Table 1.

**Table 1:** Aggregate data for the pre-mentorship (July 1, 2018—Sept 30, 2018) and post-mentorship (July 1, 2019—Sept 30, 2019) periods reported by PEPFAR-supported health facilities in four Zambian provinces (Eastern, Western, Southern, Lusaka).

	Pre-mentorship period (July 1, 2018—Sept 30, 2018)	Post-mentorship period (July 1, 2019—Sept 30, 2019)	Absolute Difference	*p-value*
Number of HIV tests performed	878,948	565,226	(313,722)	
Number of positive HIV tests	37,206	38,555	1,349	
**Proportion of people who had a positive HIV test result out of those tested for HIV**	**4.2%**	**6.8%**		***p < 0*.*001***
Number HIV tests performed on index contacts	35,386	53,859	18,473	
Number of positive index contact s	4,115	9,091	4,976	
**Proportion of people who had a positive HIV test result out of the total number of index contacts tested for HIV**	**11.6%**	**16.9%**		***p < 0*.*001***
Number of PLHIV currently on ART	492,613	521,775	29,162	
Number of PLHIV eligible for VL testing	452,239	497,560	45,321	
Number of PLHIV receiving VL test	184,488	368,801	184,313	
**VL coverage among PLHIV**	**40.8%**	**74.1%**		***p < 0*.*001***
Number of VL tests with suppressed viral load	156,383	334,453	178,070	
**Viral load suppression**	**84.8%**	**90.1%**		***p < 0*.*001***

^1^ Chi-square tests were used to compare frequencies.

## Discussion

Various models of mentorship, including mentorship provided by PLHIV themselves, have demonstrated impact in different settings [6]. Implementation of Zambia’s government led, national HIV clinical mentorship program aligned with progress toward the UNAIDS 90/90/90 targets in the four selected provinces. Program data indicated that rates of case-finding, index-case testing, HIV testing efficiency (i.e. reduction of unnecessary testing), ART initiation, viral load testing coverage, and viral load suppression significantly improved during the implementation period. In particular, the decrease in the number of HIV tests pre and post program, coupled with an increase in the number of total positive cases identified suggest that risk screening tool implementation contributed to testing efficiency implemented by the mentors in facilities. These results justify continued support for the mentorship program and make a case for implementation of similar programs in other settings.

To achieve these results, a “team of teams” approach, with solutions derived not from a hierarchical dictatorship, but rather from more complex multi-directional relationships, were critical to program success [14]. More experienced public health managers at CDC and Ministry of Health provided onsite and remote sessions on evidence-based innovations to mentors who in turn provided context to the strategies and mentorship to facility staff [4, 14, 15]. Key aspects of Zambia’s national HIV clinical mentorship program were that it was government led, which ensured authority and sustainability; incorporated video-conferencing technology, which enabled mentors to interact with mentees on a regular basis, and that there was a commitment to a continuous quality improvement methodology, which led to innovation and improvement, rather than just performance monitoring. Exceptional performance was recognized by awarding mentor’s mid-year certificates of achievements. We also recognized that accountability of mentors was critical, such that poor performance as indicated by lack of visitation to assigned sites and lack of persistence in applying continuous quality improvement methodology to achieve results was addressed through performance management improvement plans.

The PEPFAR model of working through international and non-governmental implementing partners enabled countries to rapidly scale up ART in the initial phase of the HIV response. In the current phase of the response, there is a greater focus on ensuring country ownership in order to promote sustainability while maintaining epidemic control [16]. Zambia’s national HIV clinical mentorship program is an example of an effort to develop local ownership as it helps to not only establish a capable workforce that can manage both quality for the care individuals, but also quality of programs at sites, and ultimately a successful national HIV program that utilizes a public health approach. However, as additional evidence in the optimal management of HIV and new public health approaches to epidemic control continues to emerge, even though mentors when hired were clinicians in their own right and may even have public health degrees, a key lesson learned is that mentors need ongoing mentoring, which requires significant resources. Zambia’s national HIV clinical mentorship program was the recipient of significant investment from the national ministry of health and the U.S. government, which is consistent with training and mentorship programs in epidemiology, clinical services and non-clinical health systems in the region [17–19]. Not everyone innately has qualities of a good mentor, including a willingness to spend the time to share knowledge and skills; and a genuine drive to see people succeed [7]. Specifically, for the 27 mentors, 10 CDC and 8 Ministry of Health staff provided regular mentorship and supervision. Additionally, annual trainings and quarterly retreats were held to review principles and styles of mentorship, role-play of common mentorship scenarios encountered by the mentors along with a review of current HIV practice guidelines and reflect on its application in their assigned districts and sites. While the up-front investment is substantial, the mentors advance and provide the same training and mentorship to upcoming mentors thus contributing to the building of national capacity poised to take-over management of the HIV program in Zambia from international and non-governmental organizations funded by the U.S and other donors. The success of this program will indicate that Zambia’s HIV clinical mentorship program leveraged donor funding to contribute towards building a sustainable system for delivery of HIV services going forward.

There are some limitations to our analysis; the improvements seen in the post-mentorship period could be due to improved data quality at the site level from implementation of a continuous quality improvement methodology by mentors who systematically reviewed and queried site data on a weekly and continuous basis, and cannot be attributed fully to improvement in service provision. Also, the effects of age and gender of individual mentors, seniority or specific cadre were not analyzed with respect to outcomes. However, anecdotally, mentors perceived physician mentors were viewed as more credible mentors to other physicians than lower cadres such as nurses. So, they would often rely on the support of other physician mentors when approaching other physicians. Additionally, it may not be possible to attribute all the improvements in the monitored indicators seen in the four provinces to the mentorship program, it is plausible that other factors at the individual, societal, structural and policy levels by the Ministry of Health and other implementing partners working on HIV in the provinces and not necessarily an intervention implemented contributed significantly to these achievements. However, to our knowledge, no new initiatives or interventions were implemented in the four provinces during the evaluation period that can fully explain the significant improvements in case finding, treatment coverage and viral load testing and suppression.

## Conclusions

Sound clinical knowledge and practice combined with public health practices in real-time, with continuous quality improvement as the backbone can enable program impact at facility and provincial level. To our knowledge, this is the first published report of a government-led national HIV clinical mentorship program in sub-Saharan Africa that aligned with significant improvement in key HIV programmatic indicators. Implementation of this approach should be considered in other resource limited settings.
